# The Role of Calcified Nodules in Acute Coronary Syndrome: Diagnosis and Management

**DOI:** 10.3390/ijms26062581

**Published:** 2025-03-13

**Authors:** Odysseas Katsaros, Marios Sagris, Paschalis Karakasis, Nikolaos Ktenopoulos, Stergios Soulaidopoulos, Panagiotis Theofilis, Anastasios Apostolos, Andreas Tzoumas, Nikolaos Patsourakos, Konstantinos Toutouzas, Konstantinos Tsioufis, Dimitris Tousoulis

**Affiliations:** 1 School of Medicine, National and Kapodistrian University of Athens, ‘Hippokration’ General Hospital, 11527 Athens, Greece; odykatsaros@gmail.com (O.K.); masagris1919@gmail.com (M.S.); nikosktenop@gmail.com (N.K.); soulaidopoulos@gmail.com (S.S.); panos.theofilis@hotmail.com (P.T.); anastasisapostolos@gmail.com (A.A.); ktoutouz@gmail.com (K.T.); ktsioufis@gmail.com (K.T.); 2 Department of Cardiology, “Tzaneio” General Hospital of Piraeus, 18536 Piraeus, Greece; drpats@yahoo.gr; 3 Second Department of Cardiology, Aristotle University of Thessaloniki, Hippokration General Hospital, 54124 Thessaloniki, Greece; pakar15@hotmail.com; 4 Division of Cardiovascular Health and Disease, University of Cincinnati Medical Center, Cincinnati, OH 45219, USA; andreastzoumas@hotmail.com

**Keywords:** coronary calcification, calcified nodules, acute coronary syndrome, percutaneous coronary intervention, vascular imaging, optical coherence tomography, intravascular ultrasound, atherectomy

## Abstract

Calcified nodules (CNs) are increasingly recognized as critical contributors to the pathophysiology of acute coronary syndrome (ACS). This review provides a comprehensive synthesis of the recent literature, focusing on the prevalence of CNs, their underlying mechanisms, and their implications for the clinical management of coronary artery disease (CAD). CNs are characterized by unique pathophysiological processes, and the diagnosis and treatment of CNs during percutaneous coronary interventions (PCIs) underscore the importance of advanced intravascular imaging techniques, such as optical coherence tomography (OCT) and intravascular ultrasound (IVUS), for precise identification and prognostic evaluation. Current therapeutic strategies aim to modulate CN characteristics, enhance arterial wall stability, and reduce the risk of ACS and sudden cardiac death. This review highlights the impact of CNs in ACS, the role of intravascular imaging in diagnosis, and the importance of targeted interventions to improve clinical outcomes, as by bridging diagnostic insights with emerging atherectomy modalities, this review also seeks to advance the understanding and management of CNs in PCI, fostering improved patient outcomes.

## 1. Introduction

Coronary artery disease (CAD) is a leading cause of death globally, whereas in the United States, it is responsible for one-third of all fatalities in individuals aged over 35 years [1]. Acute coronary syndrome (ACS) is an umbrella term used to cover a spectrum of coronary artery diseases, including unstable angina pectoris (uAP), ST-elevation myocardial infarction (STEMI), and non-ST-elevation myocardial infarction (NSTEMI). More recently, it has been suggested that ACS should be considered a progressive atherothrombotic disease rather than an abrupt event. ACSs are a major contributor to sudden cardiac death (SCD). ACS presents with diverse clinical manifestations, all of which result from reduced blood flow to the myocardium to varying degrees [2].

Acute luminal thrombosis within the coronary artery is a key factor in ACS. Among the primary causes, plaque rupture (PR) accounts for 65% of cases, particularly in men under 50, followed by plaque erosion (PE), which is responsible for 30% of cases and more common in women under 50. Calcified nodules (CNs), although the least frequent cause, account for 5% of cases [3,4]. However, in recent years, the role of calcified nodules in ACS has garnered increasing attention. CNs are distinct calcified structures within the coronary arteries that can lead to luminal narrowing and subsequent ischemic events. They can take on various forms, ranging from small discrete spots to large protruding lesions. Their formation involves a complex interplay of biological processes, including inflammation, osteogenic transformation, and matrix remodeling [5]. Identifying these lesions on invasive coronary angiography can be challenging; however, intracoronary imaging, such as optical coherence tomography (OCT) and intravascular ultrasound (IVUS) are highly sensitive and specific imaging techniques that offer detailed qualitative and quantitative information about underlying plaque morphology, including the detection of coronary calcium, and have detected CNs in approximately 8% of ACS cases [6,7].

This narrative review was based on a literature search in PubMed from 2010 to 2024 using the keywords ‘calcified nodules’, ‘acute coronary syndrome’, and ‘intravascular imaging’ and seeks to offer a comprehensive synthesis of the latest literature regarding the role of calcified nodules (CNs) in acute coronary syndrome (ACS). Specifically, it examines the prevalence of CNs, their underlying pathophysiological mechanisms, and the challenges they present in the clinical management and treatment of coronary artery disease (CAD). Furthermore, it highlights the pivotal role of intravascular imaging in the accurate diagnosis and prognostic assessment of CNs, while also discussing current strategies aimed at modifying CNs to enhance arterial wall stability and mitigate the incidence of ACS and sudden cardiac death.

## 2. Prevalence and Risk Factors

In their analysis of CN lesions from an autopsy registry, Torii et al. reported that the average age of patients was 70 years, with a significant prevalence of diabetes and chronic kidney disease. CNs were evenly distributed between genders, with 61.5% of nodules located in the right coronary artery (RCA), primarily within its mid-section (56%) [8]. Sugane and colleagues, using intravascular ultrasound (IVUS) to identify CNs, identified them in 5.3% of ACS patients and 5.2% of culprit lesions. Patients with CNs were more likely to exhibit CAD risk factors such as hypertension (*p* = 0.005), chronic kidney disease (*p* < 0.001), maintenance hemodialysis (*p* < 0.001), and a history of prior PCI (*p* < 0.001). They were less likely to be smokers (*p* = 0.04) and more frequently presented with unstable angina pectoris (uAP) (*p* = 0.04). Concerning medication use at discharge, CN subjects were less likely to receive a statin (83% vs. 95%, *p* = 0.01), while there were no significant differences in the use of other medications. Baseline low-density lipoprotein cholesterol (LDL-C) levels were lower in CN patients (2.6 ± 0.9 vs. 3.1 ± 0.9 mmol/L, *p* = 0.003), but their one-year levels were comparable between the two groups (2.1 ± 0.7 vs. 2.0 ± 0.5 mmol/L, *p* = 0.37). Throughout the follow-up period (median = 1304 days), the presence of CNs was linked with an increased risk of major adverse cardiovascular events (MACEs) (HR = 7.68, 95% CI = 4.61–12.80, *p* < 0.001), the recurrence of ACS (HR = 12.32, 95% CI = 6.05–25.11, *p* < 0.001), and target lesion revascularization (TLR) (HR = 10.48, 95% CI = 5.80–18.94, *p* < 0.001). These cardiac risks associated with CNs remained consistent across both Cox proportional hazards model analyses (MACE: *p* < 0.001, ACS recurrence: *p* < 0.001, TLR: *p* < 0.001) and propensity score-matched cohort analyses (MACE: *p* = 0.002, ACS recurrence: *p* = 0.01, TLR: *p* = 0.005). Notably, over 80% of TLR instances at the CN lesion were attributed to its re-appearance within the implanted drug-eluting stent (DES) [9].

Nishiguchi et al. identified CNs using pre-PCI OCT, reporting a 4.5% prevalence of CNs. Patients with CNs were generally older (*p* < 0.01) and more often female (*p* < 0.01). Hypertension (*p* < 0.01) and hemodialysis (*p* < 0.01) were also more common in CN patients compared to those without CNs. During the mean follow-up of 25.4 months, 19 cardiac deaths occurred (1 in the CN group, 18 in the non-CN group), alongside 15 non-cardiac deaths (3 in CN and 12 in non-CN patients). Kaplan–Meier survival analysis indicated significantly lower overall survival in patients with CNs (*p* < 0.05), although MACE rates were similar between the groups (*p* = 0.42) [10]. Similarly, Lee and colleagues examined new culprit lesions in patients (48% of whom had ACS) who underwent OCT prior to PCI. CNs were observed in 4.2% of all lesions, predominantly located in the ostial or mid-RCA. In a multivariable model, hemodialysis (*p* = 0.04), in-lesion angiographic Δ angle (*p* < 0.001), and maximum calcium arc by OCT (*p* < 0.001) were significantly associated with the presence of CNs. Comparing CNs in patients with ACS versus stable angina presentation revealed a smaller minimum lumen area (1.04 mm^2^ [first quartile, third quartile: 0.69, 1.26] vs. 1.61 [first quartile, third quartile: 1.03, 2.06] mm^2^; *p* = 0.02) alongside a higher incidence of thrombus (82.4% vs. 20.0%; *p* < 0.001) in CN lesions associated with ACS presentation. In lesions with severe calcification (maximum calcium arc > 180), 30% of ACS culprit lesions contained a CN, and the presence of CNs was independently associated with ACS presentation regardless of other vulnerable plaque morphologies [11].

Kobayashi et al. later sought to elucidate the clinical characteristics and outcomes of CNs, PR, and PE in ACS patients, as identified by OCT. They found that the prevalence of CNs, PR, and PE was 6%, 45%, and 41%, respectively. Patients with CNs were older (median 71 vs. 65 years; *p* = 0.03) and had a higher incidence of diabetes (71 vs. 35%; *p* = 0.002) compared to those without CNs. In OCT findings, lesions with CNs exhibited a smaller distal reference lumen cross-sectional area (median 4.2 vs. 5.2 mm^2^; *p* = 0.048) and post-intervention minimum lumen cross-sectional area (median 4.5 vs. 5.3 mm^2^; *p* = 0.04) than those without CNs. Kaplan–Meier survival curves indicated that the 500-day survival without TLR was lower (*p* = 0.011) for patients with CNs (72.9%) compared to those with PR (89.3%) or PE (94.8%) [6]. Prati et al. also observed that the presence of calcified nodules in non-culprit coronary plaques was also associated with worse clinical outcomes, including cardiac mortality and ACS in the target vessel [12].

## 3. Pathophysiologic Mechanisms and Plaque Characteristics

Previous studies have demonstrated that CNs are distinct calcified structures within the coronary arteries that can lead to luminal narrowing and subsequent ischemic events. Their formation is driven by a complex interplay of biological processes, including inflammation, osteogenic transformation, and matrix remodeling. Chronic inflammation within atherosclerotic plaques triggers the release of pro-inflammatory cytokines and growth factors, which stimulate the migration and differentiation of vascular smooth muscle cells (VSMCs) into osteoblast-like cells. These cells promote the deposition of calcium phosphate crystals, resulting in the development of calcified nodules. Pro-inflammatory cytokines such as IL-6, TNF-α, and MCP-1 contribute to calcification and CN formation by promoting vascular smooth muscle osteogenic differentiation and matrix remodeling. These cytokines facilitate the calcification process by inducing osteogenic gene expression and altering extracellular matrix composition. It is worth mentioning as well, that while obesity is recognized as a pro-inflammatory state, current evidence does not establish a direct mechanistic link between adipocytes and the formation of calcified nodules in ACS. However, future research exploring this relationship may provide further insights. Additionally, matrix remodeling, characterized by the altered expression of matrix metalloproteinases and their inhibitors, further contributes to this process [7,13,14,15,16,17].

A recent study provided new insights into CN morphology by utilizing light microscopy and microCT, exploring the progression of lesions that lead to CN development—a plaque morphology which is less frequently associated with coronary thrombosis [8]. Torii et al. found that most CNs were observed in the proximal to mid-RCA and at the left main trunk (LMT) bifurcation. These anatomical regions are known to experience excessive torsion or hinge motion during the cardiac cycle, while LMT bifurcation segments often harbor larger necrotic cores (NCs) [11,18,19,20]. In these areas, Torii’s team found that eccentric calcification is flanked by proximal and distal plaques with heavy or concentric calcification. The hypothesis that CN formation is associated with a lack of structural components, such as collagen, parallels the phenomenon of strut fractures observed in drug-eluting stents. These fractures occur more frequently in segments adjacent to “overlapping” regions, where the stiffest areas (overlapping stents) lie next to more pliable, non-overlapped stented segments, often the fracture sites [21,22]. These findings suggest that heavily calcified coronary segments directly adjacent to more flexible regions are more susceptible to external mechanical forces due to greater movement of the coronary artery during the cardiac cycle, leading to CN formation. This trial assumes that similar mechanisms likely occur for both CNs and nodular calcification areas [8]. Supporting this, Lee et al. demonstrated that lesions with nodular calcification exhibit a greater change in the angiographic angle between systole and diastole [11]. Furthermore, in this study, picrosirius red staining revealed an absence of collagen fibers within areas of nodular calcification, similar to late-stage NC. Torri and colleagues hypothesize that calcified CNs are an extension of fragmented NC calcification rather than the hard, sheet-like calcification associated with collagen-rich fibrous tissue.

Also, calcium fragmentation leading to nodule formation likely causes intraplaque hemorrhage by damaging surrounding capillaries and arterioles, resulting in clot formation involving accumulated fibrin and red blood cells. Hemosiderin deposition and macrophage infiltration may also be observed, depending on the duration of the CN. Intraplaque hemorrhage is seen in 40% of culprit CN lesions, suggesting that capillary breaks occur during calcium fragmentation. While this study cannot confirm a predisposition to thin fibrous cap disruption, the mechanical force exerted by calcified fragments likely causes the discontinuity of the overlying cap, along with a loss of surface endothelium and the formation of an overlying platelet/fibrin thrombus [8]. In the subsequent analysis of three patients who underwent microCT, longitudinal imaging provided further evidence of CN formation mechanisms. Although thrombus presence is a key factor for detecting CNs in OCT and IVUS, the clinical definition of CN recognized by imaging devices has been inconsistent regarding whether thrombus attachment is essential. Consequently, most studies report nodular calcification with an intact fibrous cap, commonly seen in heavily diseased coronary, peripheral, and carotid arteries. In the study’s sudden coronary death (SCoD) cases, surface thrombus attachment is a prerequisite for identifying CN, which is recognized as a rare cause of acute coronary thrombus [13].

## 4. Intravascular Imaging in CNs

It has already been mentioned that intravascular imaging (OCT and IVUS) constitutes the “fourth pillar” of CN detection, characterization, as well as intra-procedurally. IVUS is a highly sensitive and specific imaging technique that delivers detailed qualitative and quantitative insights into plaque morphology, including the detection of coronary calcium [23]. Five types of CNs identified by IVUS have been defined:

Type 1: An eccentric calcified nodule without calcification on the opposite side.

Type 2: An eccentric calcified nodule with broad (≥180° arc) superficial calcification on the opposite side.

Type 3: An eccentric calcified nodule with narrow (<180° arc) superficial calcification on the opposite side.

Type 4: Multiple calcified nodules within the lumen.

Type 5: A calcified nodule with visible luminal thrombus [24].

OCT is another effective method for identifying calcified nodules, characterized by fibrous cap disruption over a calcified plaque with protruding calcification, superficial calcium, and significant calcium proximal and/or distal to the lesion [7]. According to the criteria outlined in the OCT consensus document, CNs were identified by the presence of single or multiple regions of calcium protruding into the lumen, often forming sharp, jutting angles [25]. OCT can further—unlike standard resolution IVUS (we look forward to the new studies using high-definition IVUS)—differentiate an eruptive CN from a non-eruptive NC [12,26]. However, despite those recent advancements [7,11,27,28,29,30,31], challenges remain in achieving higher resolution for more the detailed characterization of CNs. The primary challenge in intravascular imaging lies in developing a reliable tool that can accurately differentiate between various pathological features with high sensitivity and specificity. Such a tool must enable the precise detection of plaque structure and composition, as well as predict future cardiovascular events. OCT has an advantage over IVUS and computed tomography angiography in identifying thrombus and the overlying PE, as PE typically involves less remodeling or thin-cap fibrous atheroma. However, OCT has limitations such as shallow image acquisition depth, the need for thrombus aspiration before imaging, and the requirement for contrast injection. Additionally, operator expertise is essential for both the functional interpretation of OCT and distinguishing pathological findings. The routine use of OCT is challenging, and the expertise required is currently limited to a few specialists.

The NIRS-IVUS imaging system was introduced as a dual-functionality device combining IVUS and near-infrared spectroscopy (NIRS) to detect lipid content within the arterial wall and plaques [32]. The Lipid Core Burden Index (LCBI) quantifies the lipid content within a given artery. Preliminary studies have shown higher LCBI content in the culprit lesions of STEMI [33]. Other studies have linked high LCBI in non-culprit lesions to future cardiovascular events. The Lipid-Rich Plaque (LRP) study was the first and largest prospective study to demonstrate that a maximum 4 mm LCBI (maxLCBI4 mm) of over 400 in a non-culprit lesion is associated with a higher risk of future cardiovascular events at both the patient and lesion level [34]. In an effort to assess whether NIRS-IVUS can distinguish between PE, PR, and CN, Terada et al. conducted a cross-sectional study of STEMI patients, using OCT as a reference standard. Their analysis revealed significant differences in NIRS-measured maxLCBI4 mm across OCT-derived PR, PE, and CN, with the highest maxLCBI4 mm observed in PR, followed by CN and PE. By evaluating plaque cavity, convex calcium, and maxLCBI4 mm, the authors concluded that NIRS-IVUS can accurately distinguish PR, PE, and CN. The remarkable accuracy between NIRS-IVUS and OCT in identifying these key morphological features is noteworthy [35].

In the era of artificial intelligence (AI), it is worth mentioning that emerging AI-driven imaging techniques and hybrid modalities, such as OCT-NIRS, may improve CN detection and risk stratification. AI algorithms are being developed to enhance the interpretation of intravascular imaging by automating plaque classification, detecting microcalcifications, and predicting lesion instability with greater accuracy than traditional methods. Deep learning models trained on large-scale imaging datasets have shown promise in differentiating between various plaque morphologies, potentially improving early CN identification and guiding intervention strategies. Thus, hybrid imaging techniques integrating AI-based OCT and IVUS analyses may further optimize decision-making in percutaneous coronary interventions.

## 5. Revascularization Difficulties

CNs present significant challenges in revascularization. There is a higher incidence of strut malposition, particularly at the nodule shoulders, due to the metal alloy’s limitations in adapting to this extreme geometry, as well as stent eccentricity and underexpansion [36]. Optimal results may not be achievable despite aggressive post-dilation, which can increase the risk of complications. In the era of drug-eluting stents (DESs), lesions with CNs are expected to negatively impact PCI outcomes [37]. TLR is more often required following PCI for lesions with severe calcification compared to those without, and previous studies have reported TLR rates ranging from 20.0% to 38.0% after 2 years, especially in the group with eruptive CNs [6,37,38,39,40]. Morofuji and colleagues found that CNs were present in half of the severely calcified lesions requiring rotational atherectomy and were associated with worse adverse outcomes after a 5-year follow-up, while Sugane et al.—as mentioned above—reported that more than 80% of TLR at the CN lesion was due to the recurrence of CNs within the implanted DES [9,37]. Their findings suggest that CNs continue to protrude even after stent placement and in-stent restenosis at CN lesions has been described in a pathohistological investigation as CNs protruding through the stent struts and thrombus or neointima calcification within the implanted stent [41,42]. Sato et al., in a recent study, found a 2-year cumulative rate of target lesion failure (TLF) primarily caused by clinically indicated TLR and indicated that eruptive CN morphology has a different impact on long-term clinical outcomes compared to non-eruptive CN morphology [43]. Another OCT study demonstrated that patients with eruptive CNs had a significantly higher 2-year incidence of cumulative major adverse cardiovascular events (MACEs) compared to the calcified protrusion and superficial calcific sheet groups, suggesting that eruptive CNs in culprit lesions in ACS patients more frequently impact clinical outcomes after PCI [44].

Until recently, no systematic studies utilizing intracoronary imaging modalities had demonstrated the influence of CNs in non-culprit lesions. However, Xu et al. reported that CNs in the non-culprit lesions of ACS patients resulted in better clinical outcomes over a 3-year follow-up period [27]. Consistently with those results, Wu and colleagues—investigating ACS patients performing IVUS to evaluate non-culprit lesions—found that there were no deaths, cardiac arrests, or myocardial infarctions in the CN group. Surprisingly, while one pathology group has described culprit CNs as a rare cause of coronary thrombosis, non-culprit CNs are thought to represent precursor lesions similar to thin-cap fibroatheromas (TCFs). It is important to note that CNs do not always cause thrombosis, just as TCFs do not always cause plaque rupture (PR). In the same study, they reported that the CN group had fewer non-culprit lesion MACEs compared to the non-CN group. They hypothesized that CNs may develop from plaque rupture, thrombosis, and subsequent healing, potentially stabilizing the non-culprit lesion rather than contributing to adverse outcomes [45]. Additionally, operators must consider that the combination of tortuosity, nodules, and hinge motion may hinder device delivery and increase the risk of stent fracture and target lesion failure [26].

In the near future, advancements in bioresorbable scaffolds and targeted plaque modification strategies may optimize treatment outcomes for patients with CN-related ACS.

## 6. Intra-Procedural Modification of Calcified Nodules

**Figure 1:**
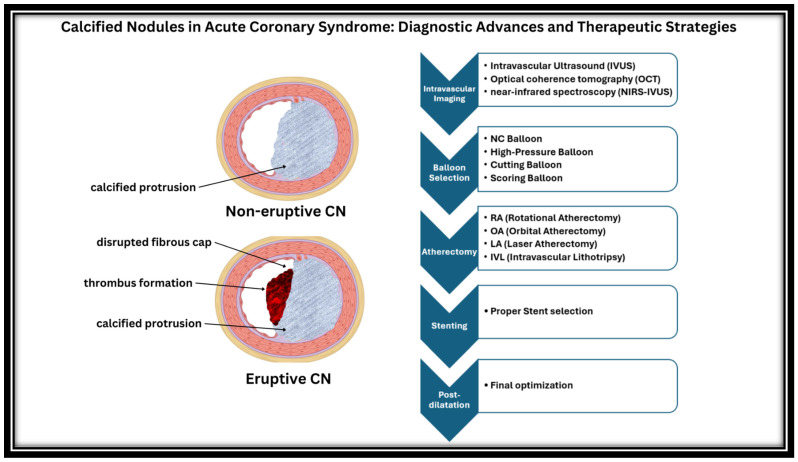
Algorithm for the management of calcified nodules during PCI.

The procedural preparation and modification of vessels with CNs is crucial (Figure 1). Balloon dilation primarily works by eccentrically expanding the healthy vessel wall opposite the nodule, but it carries a higher risk of dissection and perforation and has only a marginal effect on the nodule itself.

### 6.1. Non-Compliant Balloons (NC)

NC balloons are commonly used to treat mildly to moderately calcified coronary lesions by achieving more consistent stent expansion than semi-compliant balloons. However, in cases of severe calcification, the balloon’s expansion can become irregular, increasing the risk of complications such as coronary dissection, perforation, or balloon rupture due to high pressure at the edges. Despite these risks, NC balloons are valuable when used after atherectomy to ensure adequate plaque modification before stenting [46,47].

### 6.2. High-Pressure Balloons

The super high-pressure balloon (OPN NC, SIS Medical, Frauenfeld, Switzerland) is designed to withstand extremely high pressures (up to 35 atm), which allows it to successfully dilate calcified lesions that are resistant to conventional NC balloons. In a retrospective study of 326 patients, this balloon achieved a high success rate (over 90%) in treating non-dilatable calcified lesions, though coronary rupture occurred in a small number of cases [48,49,50,51,52]. This balloon is also used for post-dilation to optimize stent expansion [53].

### 6.3. Cutting Balloons

Cutting balloons (FlexTome and Wolverine, Boston Scientific, Marlborough, MA, USA) are NC balloons with small blades attached to their surface. These blades create precise incisions in calcified plaques, aiding in stent expansion, particularly in challenging lesions such as those in the ostium or with in-stent restenosis (ISR) [54,55]. Although cutting balloons have demonstrated superior lumen gain compared to standard balloons, they are associated with a slightly higher risk of coronary perforation [54,56,57,58,59]. Recent advancements in cutting balloon design have improved their deliverability, although complications like blade entrapment and coronary artery perforation remain concerns [60].

### 6.4. Scoring Balloons

Scoring balloons (AngioSculpt, Philips, San Diego, CA, USA; Scoreflex, OrbusNeich, Hong Kong, China; Chocolate XD, Teleflex, Wayne, PA, USA; NSE Alpha, B. Braun, Melsungen, Germany; Lacrosse NSE, Asomedica, Minsk, Belarus) are semi-compliant and feature scoring elements on their surface that focus force on the calcified plaque during inflation. These balloons offer easier deliverability and a lower risk of vessel wall injury than cutting balloons, while still providing effective luminal expansion. Despite the absence of direct comparative trials, scoring balloons are generally considered an alternative to cutting balloons, particularly in cases where vessel injury risk is a concern. Preliminary studies have shown scoring balloons to be effective in modifying calcium in severely calcified lesions [46,61,62,63,64,65,66,67].

**Concerning 6.1–6.4**: The above-mentioned methods refer to calcified lesions or severely calcified lesions, which, as mentioned in this review, have unique particularities and difficulties that should be encompassed to the already challenging treatment of calcified lesions. A balloon-only approach may be constrained by the eccentric expansion of the balloon, which might not generate sufficient force to effectively modify CNs surrounded by severe calcification. This limitation could lead to significant stent underexpansion and asymmetry [46,53]. Specialty balloons, such as cutting or scoring balloons, may offer theoretical advantages by enabling controlled and uniform lesion dilation while minimizing balloon slippage in the presence of eccentrically protruding CNs. However, their effectiveness in treating CNs has not been thoroughly investigated.

### 6.5. Rotational Atherectomy (RA)

Rotational atherectomy (RA), including devices like Rotablator and RotaPro from Boston Scientific, employs a high-speed, diamond-tipped burr to mechanically ablate hard, calcified atheroma while sparing more pliable, non-calcified tissue. This high-speed rotation enlarges the lumen, creates a smoother luminal surface, and reduces plaque rigidity, which facilitates balloon predilatation and enhances stent expansion [68]. Current guidelines recommend RA to improve procedural success in fibrotic or heavily calcified lesions (class 2 a, level of evidence B) [69].

RA has been extensively studied and is regarded as the gold standard for modifying severely calcified lesions before stenting, particularly in lesions that are resistant to balloon crossing [70,71,72,73,74,75,76,77,78,79,80]. However, RA carries risks such as coronary dissection [70,71,72,73,74,75,76,77,78,79,80], perforation, and transient slow or no-reflow events, often related to the complexity of the lesions treated [81,82]. Despite these risks, RA is recommended for improving procedural success in fibrotic or heavily calcified lesions [83,84,85,86,87]. Contemporary RA techniques and the use of smaller burrs have reduced complication rates, particularly in high-volume centers with experienced operators [81,88]. While the primary benefit of RA is facilitating successful PCI in severely calcified lesions, current data do not conclusively demonstrate long-term clinical benefits.

### 6.6. Orbital Atherectomy

Orbital atherectomy (OA), with the Diamondback 360 from Cardiovascular Systems Inc., St. Paul, MN, USA, received FDA approval in 2013 for treating severely calcified coronary lesions. This device uses an eccentrically mounted, diamond-coated crown that ablates calcified plaque through elliptical motion [89]. This method allows for the selective ablation of non-flexible, calcified tissue while sparing the more pliable vessel wall. OA offers several advantages over RA, including the reduced risk of slow/no-reflow events and thermal injury [82,84,90]. Clinical studies, such as the ORBIT series, have shown OA to be effective in treating severely calcified lesions with low rates of major adverse cardiovascular events (MACEs) [89,91,92,93,94,95,96]. However, randomized controlled trials directly comparing OA to RA are still needed. 

**Concerning 6.5–6.6**: Patients with CNs consistently demonstrate poorer clinical outcomes following RA-assisted PCI compared to those without CNs [24,37]. Additionally, RA has not been proven to lower the risk of ischemia-driven target vessel revascularization in these patients [24,97]. This may indicate that RA does not always adequately modify eccentric CNs, potentially due to guidewire bias caused by the nodules and adjacent calcium sheets, which can deflect the centrally mounted burr away from the calcium. While larger burrs might improve the debulking of eccentric CNs, their use is associated with a higher risk of complications. In contrast, OA may offer theoretical advantages over RA for treating CNs, as its eccentrically mounted crown and circumferential shaving mechanism allow for more consistent modification. However, evidence on the effectiveness of OA in treating CNs remains limited. Furthermore, the degree of debulking achieved by either OA or RA may be modest, given the typically thick nature of CNs and their occurrence in large vessels. Despite limited debulking, atherectomy might still be valuable or necessary to facilitate equipment delivery; in the absence of OA, larger RA burrs should be considered.

### 6.7. Intravascular Lithotripsy (IVL)

Adapted from lithotripsy technology for treating kidney stones, IVL (Shockwave C2 coronary IVL, Shockwave Medical, Santa Clara, CA, USA) utilizes shockwaves to fracture calcified plaques, thereby improving vessel compliance and stent expansion. The IVL balloon catheter generates high-pressure shockwaves that selectively fracture calcified areas within the vessel wall while sparing elastic tissue [98]. Initial studies have demonstrated IVL’s safety and efficacy, with high procedural success rates and minimal complications [99,100,101,102]. IVL has been shown to be particularly effective in managing severely calcified coronary lesions, reducing stenosis significantly with minimal risks of dissection or perforation [103,104].

In light of this, unlike atherectomy, IVL is not influenced by guidewire bias and delivers energy circumferentially, allowing for uniform calcium disruption at both the level of CNs and the adjacent segments of calcified plaques. Additionally, IVL can address deep calcium surrounding CNs, which is a critical factor in limiting stent expansion [105,106,107,108,109,110]. Recent findings from the Disrupt CAD OCT substudies demonstrated that IVL effectively treats CNs, showing no significant differences in residual stenosis, stent expansion, acute gain, or target lesion failure at 2 years between CNs and non-CNs treated with IVL [111].

### 6.8. Laser Atherectomy

Laser atherectomy, specifically the Excimer Laser Coronary Atherectomy (ELCA) with the CVX−300 from Philips, San Diego, CA, USA, has been utilized for over 20 years as an alternative to BA. This technique employs photoablation to modify plaque. The device emits pulses of short-wavelength, high-energy ultraviolet light, which vaporizes water, dissociates carbon bonds, and causes molecular vibrations, resulting in plaque obliteration and enhanced luminal expansion, facilitating the treatment of challenging lesions, including balloon-uncrossable or undilatable lesions and chronic total occlusions [70,112,113,114,115,116,117,118,119,120,121]. ELCA is particularly useful for modifying calcific non-dilatable in-stent restenosis (ISR) and has demonstrated high technical and procedural success rates with low MACEs. The LAVA registry, which assessed ELCA’s use in complex coronary lesions, confirmed its efficacy and safety, particularly in de novo calcified lesions and ISR [122]. However, evidence on the effectiveness of LA in treating CNs remains very limited (Table 1).

Summarizing, Table 2 offers a comprehensive synopsis of the aforementioned pathophysiological mechanisms, diagnosis, as well as treatment strategies for calcified nodules.

## 7. Future Directions

Future research should focus on refining imaging modalities to improve the detection and characterization of CNs, particularly integrating artificial intelligence for real-time analysis. The development of high-resolution intravascular imaging and hybrid imaging techniques, such as OCT-NIRS, could enhance diagnostic accuracy and guide targeted interventions. Additionally, long-term studies assessing the natural progression of CNs and their role in non-culprit lesions could provide valuable insights into their clinical significance and optimal management strategies.

Further innovation in therapeutic approaches is necessary. Improvements in bioresorbable scaffolds and targeted plaque modification techniques hold promise for better procedural success and long-term outcomes in patients with CN-related ACS. Pharmacological advancements aimed at modulating the biological processes underlying CN formation, such as inflammation and osteogenic transformation, may offer new avenues for disease stabilization and prevention.

## 8. Conclusions

Calcified nodules represent a unique challenge in the management of acute coronary syndrome, contributing to increased risks of procedural complications and adverse cardiovascular outcomes. Intravascular imaging techniques have significantly improved the ability to detect and characterize CNs, yet limitations remain in predicting their long-term behavior. Current revascularization strategies require further optimization, particularly in addressing stent malapposition and underexpansion caused by CN morphology.

Continued research and technological advancements are crucial for improving patient outcomes. A multidisciplinary approach integrating advanced imaging, novel stent designs, and innovative therapeutic strategies will be essential in overcoming the challenges posed by CN-related ACS.

## Figures and Tables

**Table 1:** Evidence-based interventions for calcified nodules.

Technique	Mechanism of Action	Key Evidence	Advantages	Limitations/Complications	Clinical Use
**Non-Compliant Balloons**	High-pressure inflation reshapes calcified plaques.	Effective for mild/moderate calcifications but limited for severe calcifications.	Safe for mild calcifications; low cost.	Dissection and perforation risks at high pressure.	Predilation before stenting in mildly calcified lesions.
**High-Pressure Balloons**	Twin-layer technology withstands pressures up to 35 atm.	A 90% success rate in non-dilatable lesions; rare coronary rupture risk.	Effective where NC balloons fail.	Limited data; potential coronary rupture.	Treating resistant calcified lesions and optimizing stent expansion.
**Cutting Balloons**	Blades incise calcified plaque to aid dilation.	Larger lumen gain; 0.8% risk of perforation.	Precise, focused luminal gain.	Increased risk of perforation and device entrapment.	Focal calcifications, ostial lesions, and in-stent restenosis (ISR).
**Scoring Balloons**	Scoring elements concentrate force to fracture calcifications.	Safer alternative to cutting balloons; proven efficacy in ISR.	Lower dissection risk than cutting balloons.	Not effective for dense calcifications.	Moderate calcifications or ISR; safer luminal gain in eccentric nodules.
**Intravascular Lithotripsy (IVL)**	Acoustic waves fracture calcium without damaging soft tissue.	A 92.4% procedural success rate with minimal complications.	Uniform energy delivery; minimal embolization risk.	Limited deliverability in tortuous vessels.	Severe or eccentric calcifications; adjunct to atherectomy for resistant CNs.
**Rotational Atherectomy**	Diamond-tipped burr ablates calcifications, reducing rigidity.	Gold standard for severe calcifications; PREPARE-CALC trial confirms procedural success.	Effective for deep, dense calcifications.	Risk of slow/no-reflow events; operator dependent.	Severe calcifications resistant to balloon angioplasty; may facilitate device delivery in CNs.
**Orbital Atherectomy**	Elliptical crown ablates calcifications while sparing pliable tissue.	Comparable safety and efficacy to RA; no large RCTs yet.	Lower thermal injury risk than RA.	Requires further evidence; risk of distal embolization.	Treating deep, eccentric, or superficial calcium; better modification of CNs.
**Laser Atherectomy**	UV laser photoablates plaque by vaporizing water and breaking carbon bonds.	LAVA registry shows 90% technical success in undilatable ISR.	Effective for ISR and chronic occlusions.	Niche application; requires specialized equipment.	Balloon-uncrossable or undilatable lesions and ISR.

**Table 2:** Summary of pathophysiological mechanisms, diagnosis, and treatment strategies for calcified nodules.

Category	Key Features	Clinical Implications	Diagnostic Approaches	Therapeutic Strategies
**Pathophysiology**	- Chronic inflammation - Microcalcification - Endothelial disruption - Mechanical stress - Matrix remodeling	- Plaque instability - Ischemic events - Increased risk of MACE	- Optical coherence tomography (OCT) - Intravascular ultrasound (IVUS) - Near-infrared spectroscopy (NIRS)	- Targeted medical therapy (statins, anti-inflammatory agents) - Lifestyle modifications
**Isolated Calcified Nodules**	- Discrete nodular calcifications without plaque rupture/erosion	- May cause luminal narrowing and thrombus formation - Less endothelial damage	- IVUS for morphology - OCT for detailed cap structure	- Balloon angioplasty (non-compliant or high-pressure balloons) - Intravascular lithotripsy (IVL)
**Calcified Nodules with Plaque Rupture or Erosion**	- Nodular calcifications with overlying thrombus and cap disruption	- High risk of acute thrombosis - Increased PCI challenges	- OCT to assess fibrous cap disruption - IVUS/NIRS for plaque composition	- Atherectomy (rotational/orbital) - Drug-eluting stents (DESs) - IVL for deeper calcium fractures
**Therapeutic Challenges**	- Stent malapposition - Underexpansion - Recurrent target lesion revascularization (TLR)	- Poor PCI outcomes - Higher restenosis rates	- Post-PCI imaging (OCT/IVUS) to assess stent expansion and apposition	- Aggressive lesion preparation (atherectomy, scoring/cutting balloons) - Stent optimization techniques (high-pressure post-dilation, IVL)

## Data Availability

The data supporting the findings of this study are available in PubMed (https://pubmed.ncbi.nlm.nih.gov/ accessed on 19 December 2024).
